# The relationship between quality of research and citation frequency

**DOI:** 10.1186/1471-2288-6-42

**Published:** 2006-09-01

**Authors:** Pentti Nieminen, James Carpenter, Gerta Rucker, Martin Schumacher

**Affiliations:** 1 Medical Informatics Group, University of Oulu, P.O. Box 5000, FIN-90014 Oulu, Finland; 2 Institute of Medical Biometry and Medical Informatics, University of Freiburg, Stefan-Meier-Str. 26, D-79115 Freiburg, Germany

## Abstract

**Background:**

Citation counts are often regarded as a measure of the utilization and contribution of published articles. The objective of this study is to assess whether statistical reporting and statistical errors in the analysis of the primary outcome are associated with the number of citations received.

**Methods:**

We evaluated all original research articles published in 1996 in four psychiatric journals. The statistical and reporting quality of each paper was assessed and the number of citations received up to 2005 was obtained from the Web of Science database. We then examined whether the number of citations was associated with the quality of the statistical analysis and reporting.

**Results:**

A total of 448 research papers were included in the citation analysis. Unclear or inadequate reporting of the research question and primary outcome were not statistically significantly associated with the citation counts. After adjusting for journal, extended description of statistical procedures had a positive effect on the number of citations received. Inappropriate statistical analysis did not affect the number of citations received. Adequate reporting of the primary research question, statistical methods and primary findings were all associated with the journal visibility and prestige.

**Conclusion:**

In this cohort of published research, measures of reporting quality and appropriate statistical analysis were not associated with the number of citations. The journal in which a study is published appears to be as important as the statistical reporting quality in ensuring dissemination of published medical science.

## Background

Citation by other authors is important in the dissemination of published study findings. The attention that scientific articles get can be assessed using citation analysis. In this context, Egghe & Rousseau [1] claim that four important assumptions form the basis for all research based on citation counts. The assumptions are that: (1) citation of an article implies use of that document by the citing author, (2) citation reflects the merit (quality, significance, impact) of the article, (3) references are made to the best possible works, and (4) an article is related in content to the one in which it is cited. Thus citation counts can be regarded as one method of obtaining a quantitative expression of the utilization and contribution of a particular published paper. However, whether received citations reflect the methodological quality has been questioned [2].

Statistical methods play an important role in medical research. This is reflected in the high proportion of articles which are essentially statistical in their presentation [3,4]. The most visible aspect of this is the statistical summaries of the raw data used in the research. Medical research articles using statistical methods have always been at risk of poor reporting, methodological errors and selective conclusions [5-8]. The existence of these problems in published articles is often regarded as evidence that poor research and poor reporting quality slips through the peer review process [6,9].

The association between statistical reporting and the number of citations received is presently unclear [10]. Our aim is to investigate the extent to which authors consider the quality of the evidence when deciding which evidence to cite. We hypothesised that publications are cited for a variety of reasons, but that the effect of statistical reporting and inappropriate statistical analysis on the number of citations is minimal.

## Methods

### Set of articles

For our investigation we selected four general English-language psychiatric journals: The *American Journal of Psychiatry *(AJP), *Archives of General Psychiatry *(AGP), the *British Journal of Psychiatry *(BJP) and the *Nordic Journal of Psychiatry *(NJP). AJP and AGP are the two leading medical journals covering psychiatric research and have consistently been the top two as ranked by Garfield's impact factor (IF), while BJP is the most cited psychiatric journal outside the United States and NJP represents the large group of journals having a markedly lower IF than the other three studied here. The four journals had the following impact factors in 2004: AGP 11.207, AJP 7.614, BJP 4.175 and NJP 0.887.

All the articles published in these four journals in 1996 were supplied by the Medical Libraries in the authors' institutes. Papers were included for examination if they had been published as original research articles in 1996, reported research findings based on the systematic collection of data, and used statistical methods for data analysis. The total number of articles reviewed was 448, representing about 47% of all the articles in the four journals (N = 951). Those excluded were mostly letters (n = 287), brief reports (AJP, n = 63), reviews (n = 22) or editorials. Further details of the sample and the statistical methodology used in the articles have been published in an earlier paper [4].

### Number of citations

Each article's citations, over 9 years till April 2005, were obtained from the Web of Science databases (Science Citation Index, Social Sciences Citation Index and Arts & Humanities Citation Index) in April 2005. Self-citation was deemed to have occurred whenever the set of co-authors of the citing article shared at least one author with that of the cited one, a definition used in various recent citation studies [11]. The number of self-citations was then subtracted from the total number of citations recorded.

### Primary outcome in the evaluated articles

One reviewer (P.N.) assessed all papers to determine the primary outcome and main response variable(s) together with possible explanatory or grouping factors. The primary outcome was that which was stated in the research objectives (in the abstract or introduction) or labelled as "primary" in the methods. When no outcome met these criteria, the reviewer used his own judgment to select the outcome that was presented in the abstract, and/or the first outcome presented in the results, that appeared crucial to the final conclusions. The psychiatric sub-field and study design were also assessed. Papers that were difficult to assess were additionally reviewed by GR and MS, then jointly assessed.

To ensure consistency of evaluation (assessments), the assessor used the same detailed (manual) classification scheme for each paper, and was blind to the number of citations received.

The reliability of the evaluation was investigated by comparing the ratings of two reviewers (P.N. and Jouko Miettunen). They independently reviewed all the 448 articles in separate research projects with different research questions; however, their review protocols shared two items. For the first, 'whether data analysis procedures were completely described in the methods part of the research report', the overall agreement between raters was 90.7% and kappa coefficient for inter-rater reliability was 0.75 (95% CI 0.68 – 0.82). For the second, 'whether the statistical software used in the study was named in the report', the overall agreement was 96.9% and kappa coefficient 0.93 (95% CI 0.89 – 0.96).

### Characteristics of the statistical reporting and analysis

To evaluate the quality of reporting, the following information was obtained: (i) whether the primary research question or hypothesis was clearly stated in the report's introduction or methods section; (ii) whether sample size and data analysis procedures were described in the report's methods section, and (iii) whether the article was difficult to read due to lack of clarity about the primary response or outcome variable.

Each article was also assessed for inappropriate use of statistical methods. Specifically, each article was checked for the specific analysis errors defined by Altman [8] as 'definite errors'. These errors are related to elementary statistical techniques and included the following: (i) using a statistical test that requires an underlying normal distribution on data that are not normally distributed; (ii) using an incorrect method for repeated measurements, analyzing serial measurements independently at multiple time points and making comparisons between p-values; (iii) using a non-parametric test that requires an ordered scale on data with non-ordered categorical variable; (iv) wrong unit of analysis, confusion between tests or more tests than number of cases; or (v) other errors such as using an incorrect method for time-to-event data or using a correlation coefficient to relate change to initial value.

Studies were categorised as including insufficient or incomplete analysis if the article drew conclusions not supported by the study data, reported significant findings without a statistical test or CI, or explicitly or implicitly made comparisons between p-values. The overuse of statistical tests, defined to be present if there was no clear main hypothesis, or several sub-group analyses using the primary outcome, was also assessed.

As it is plausible that studies with larger sample sizes include more evidence, we categorised each study's sample size as small (< 50), medium (50 – 360) or large (>360).

### Statistical analysis

Box plots showed that the distribution of the number of citations received is highly positively skewed, so we use the median as a measure of location. Mann-Whitney tests, Kruskal-Wallis ANOVA and negative binomial regression were used to investigate possible associations between the number of citations and reporting quality. We adjusted for journal to control for the effect of journal visibility. The statistical significance of differences in statistical reporting and errors between the four journals was evaluated using chi-square test.

The statistical software used were the SPSS for Windows version 12.0 (SPSS Inc.) and SAS Release 9.1 (SAS Institute Inc.).

## Results

The articles in our sample came from a variety of specialties: epidemiology, clinical topics, psychopharmacology, biological topics and others. The distribution of published articles by journal and topic is shown in table 1. AJP and NJP had more clinical articles than the other two journals, BJP had more other articles (e.g. prevalence and validity studies) and AGP had more biological articles compared to other evaluated journals. The distribution of study designs was as follows: cross-sectional surveys (33.7%), cohort studies (26.8%), case-control studies (16.5%), intervention studies including clinical trials (16.7%), reliability and diagnostic studies (4.7%) and basic science studies (1.6%).

**Table 1:** Distribution of the psychiatric sub-field of the original research articles and median number of citations received by the publishing journal.

	Sub-field of psychiatry												Received citations	
				
Journal	Epidemiology		Clinical topics		Psychopharmacology		Biological topics		Others		All articles			
	N	%	N	%	N	%	N	%	N	%	N	%	Median	Range
AJP	21	15.6	51	37.8	14	10.4	25	18.5	24	17.8	135	100	33	1 – 194
AGP	21	23.2	19	21.1	13	14.4	29	32.2	8	8.9	90	100	64	7 – 297
BJP	40	21.7	40	21.7	16	8.7	20	10.9	68	37.0	184	100	20	1 – 112
NJP	8	20.5	15	38.5	5	12.8	2	5.1	9	23.1	39	100	1	0 – 7
Total	90	20.1	125	27.9	48	10.7	76	17.0	109	24.3	448		20	0 – 297

### Citation frequencies

**Figure 1:**
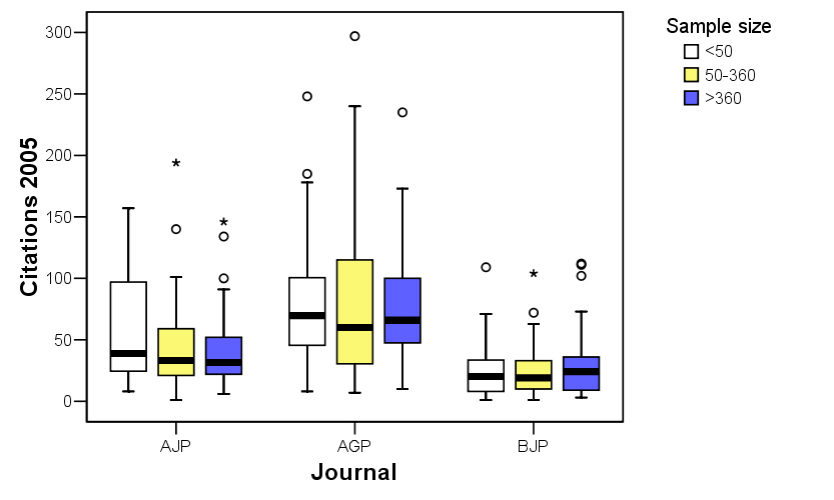
Number of citations received by the sample size in three psychiatric journals. NJP is not included due to low number of citations.

Figure 1 shows how the number of citations varies by journal and sample size. Excluding self-citations, up to April 2005 the AGP articles received a median of 64 citations while the median for those in the AJP was 33 and for those in the BJP was 20. Few references were made to articles published in the low IF journal NJP (median 1, not included in the figure 1 due to low number of citations).

### The quality of reporting

Table 2 shows the distributions of the reporting quality measures by journal. Failure to state the primary research question or hypothesis was most common defect (34.6%). Of the 448 evaluated articles, sample size was unreported in 78 (17.4%) papers. The quality of reporting was related to the journal; failure to describe the primary research question and methods was less common in the AJP and AGP.

**Table 2:** Distribution of the reporting quality variables and median of the number of citations received by the publishing journal.

	AGP			AJP			BJP			NJP			Total		P-value of chi square test
						
	N	(%)	Md	N	(%)	Md	N	(%)	Md	N	(%)	Md	N	(%)	
Research question															< 0.001
▪not stated	18	(20.0)	50.5	38	(28.1)	32	78	(42.4)	19.5	21	(53.8)	1	155	(34.6)	
▪stated	72	(80.0)	73.5	97	(71.9)	33	106	(57.6)	20.5	18	(46.2)	1	293	(65.4)	
Primary outcome															0.004
▪not stated	20	(22.2)	49.5	28	(20.7)	36	45	(24.5)	20	19	(48.7)	0	112	(25.0)	
▪stated	70	(77.8)	74	107	(79.3)	32	139	(75.5)	20	20	(51.3)	1.5	336	(75.0)	
Sample size															0.047
▪not reported	12	(13.3)	58	16	(11.9)	33.5	42	(22.8)	16	8	(20.5)	1	78	(17.4)	
▪reported	78	(86.7)	65.5	119	(88.1)	33	142	(77.2)	23	31	(79.5)	1	370	(82.6)	
Incomplete description of procedures															< 0.001
▪yes	7	(7.8)	63	21	(15.6)	**24 **^a^	66	(35.9)	**16 **^b^	19	(48.7)	1	113	(25.2)	
▪no	83	(92.2)	65	114	(84.4)	**34**	118	(64.1)	**24**	20	(51.3)	0.5	335	(74.8)	
All	90			135			184			39			448		

The statistical significance of differences of the medians between variable categories was tested with Mann-Whitney U test. The two with **p < 0.05 are in bold.**

^a ^p-value of Mann-Whitney U test is 0.020.

^b^p-value of Mann-Whitney U test is 0.039.

Table 2 also shows the median number of citations for articles in each journal by the reporting quality measures. There was not a strong association between the quality of reporting and the number of citations received by the articles. In the AGP, articles with better reporting quality received more citations, but this association was not statistically significant in any of the quality variables. Only in the AJP and BJP did 'description of statistical procedures' have a statistically significant positive association with the number of citations received (Mann-Whitney test, p < 0.05)

### Errors in statistical analysis

Table 3 compares the prevalence of statistical errors in the four journals. A total of 17 articles (3.8%) used a statistical test that requires an underlying normal distribution on data that clearly was not normally distributed; 5.8% (26 articles) used an incorrect method for repeated measurements (unpaired or independent samples); 0.9% (4 articles) used a test that requires an ordered scale on data with non-ordered categorical variable; 5.6% (25 articles) had confusion with observation units, confusion between tests or more tests than number of cases; and 0.6% (3 articles) had other errors. Inappropriate analyses seemed to be less common in the more visible journals. The total error rate of 16.7% is probably an underestimate, because often articles did not give enough information to evaluate the appropriateness of the methods they used. 31.5% (141 articles) met our criteria of overuse of statistical significance tests (i.e. they lacked a clear main hypothesis or had several sub-group analyses using the primary outcome).

**Table 3:** Distribution of the 'quality of statistical analysis' variables and median number of citations received by the publishing journal.

	AGP			AJP			BJP			NJP			Total		P-value of chi square tests
						
	N	(%)	Md	N	(%)	Md	N	(%)	Md	N	(%)	Md	N	(%)	
Inappropriate analysis															0.044
▪yes	10	(11.1)	64	20	(14.8)	33.5	33	(17.9)	25	12	(30.8)	0.5	75	(16.7)	
▪no	80	(88.9)	64	115	(85.2)	33	151	(82.1)	19	27	(69.2)	1	373	(83.3)	
Incomplete analysis															0.018
▪yes	18	(20.0)	68	30	(22.2)	31.5	39	(21.2)	20	17	(43.6)	0	104	(23.2)	
▪no	72	(80.0)	64	105	(77.8)	34	145	(78.8)	20	22	(56.4)	1	344	(76.8)	
Overuse of tests															0.195
▪yes	29	(32.2)	52	38	(28.1)	34	56	(30.4)	19	18	(46.2)	1	141	(31.5)	
▪no	61	(67.8)	66	97	(71.9)	32	128	(69.6)	22.5	21	(53.8)	1	307	(68.5)	
All	90			135			184			39			448		

The statistical significance of differences between variable categories was tested with Mann-Whitney U test. No statistically significant (p < 0.05) differences were found.

Table 3 also gives the median number of citations received by the papers in each journal by the statistical analysis variables. There is no evidence that errors in the statistical analysis of the primary outcome decreased the number of citations.

### Adjusted effects on the number of citations

An estimated multivariate negative binomial regression model for the effects of quality of statistical reporting and analysis on the number of received citations, adjusted for the publication forum, is shown in Table 4. Journal visibility is the most important predictor of citation frequency; the citation rate in the AGP is three times that in the BJP. After adjustment for journal, articles which have an inadequate description of statistical procedures have a ratio of 0.83 (95% CI 0.80 – 1.20, P = 0.048) citations per article relative to those with extended description. Other reporting quality or statistical analysis variables were not associated with citation frequency.

**Table 4:** Adjusted negative binomial regression model for the impact of statistical reporting and analysis on citation frequency.

Variable	Ratio	Standard error	95% CI Lower Bound	95% CI Upper bound	P-value
Intercept	27.25	1.08	23.24	31.95	
Journal					
▪AGP	3.11	1.11	2.53	3.82	< 0.001
▪AJP	1.77	1.10	1.48	2.11	< 0.001
▪NJP	0.06	1.21	0.04	0.08	< 0.001
▪Reference BJP	1.00				
Research question					
▪not stated	0.99	1.10	0.82	1.19	0.888
▪stated	1.00				
Primary outcome					
▪not stated	0.94	1.10	0.78	1.14	0.554
▪stated	1.00				
Sample size					
▪not reported	0.98	1.11	0.80	1.20	0.855
▪reported	1.00				
Incomplete description of procedures					
▪yes	0.83	1.10	0.69	1.00	0.048
▪no	1.00				
Inappropriate analysis					
▪yes	1.05	1.12	0.85	1.31	0.640
▪no	1.00				
Incomplete analysis					
▪yes	0.95	1.10	0.78	1.15	0.601
▪no	1.00				
Overuse of tests					
▪yes	1.026	1.10	0.86	1.23	0.778
▪no	1.00				
Dispersion parameter	1.75	1.04	1.62	1.88	

Further, in an additional analysis (suggested by a reviewer) we investigated whether there is a difference in the number of citations received by papers with (i) statistical errors that potentially affect the study results and (ii) papers with reporting failures. To this end, a combined variable "Presence of errors potentially affecting the study results" was defined using the last four variables given in Table 4. It takes the value "yes" if there is an inappropriate analysis or overuse of tests, and "no" if there is a complete description of the procedures, a complete and appropriate analysis, and no overuse of tests. In all other cases, it takes the value "undetermined". The negative binomial regression model was then with journal and this new variable as the only covariates. The results showed no evidence of an association between this new variable and citation. Arguably, this is unsurprising as this new variable effectively dilutes the association shown in Table 4.

### Sample size

Only 16 out of the 448 psychiatric articles published in the four journals in 1996 included sample size calculations, power analysis or any other justification for the sample size, contrary to the CONSORT [12] and STROBE [13] guidelines for reporting research.

Figure 1 shows the distribution of number of citations by the sample size in three of the journals. NJP is not included due to low number of citations. There was no statistically significant evidence of preferential citation of studies with large sample size (p-value of Kruskal-Wallis test > 0.05 in each journal).

## Discussion

This study investigated the association between the quality of an article's statistical reporting and analysis and the number of citations it received. In this set of articles, failing to state essential information, such as the primary research question or the primary outcome variable did not affect the number of citations the article received. However, a sufficient description of the methods used was an important factor in increasing the number of citations received in two of the four journals. Statistical errors and sample size were not associated with number of citations received. Reporting quality was associated with the journal visibility and prestige.

West and McIlwaine [14] have analyzed citation counts in the field of addiction studies. They report that there was no correlation between number of citations and expert ratings of article quality. Callaham et al [15] examined a cohort of published articles originally submitted to an emergency medicine meeting and also reported that the impact factor of the publishing journal, not the peer rating quality of the research, was the strongest predictor of citations per year. Our findings concerning the statistical quality are in line with these findings.

The importance of stating the purpose and a priori hypotheses of a research project in the report is obvious, but such a statement was often (in 34.6% of papers) missing. In these cases, the results cannot be interpreted in light of a priori hypotheses. Further, unless the research question is clearly stated, the appropriateness of the study design, data collection methods and statistical procedures cannot be judged. For other researchers to cite the paper, however, it does not appear to matter whether the initial purpose of the cited study was clear, or whether the analyses are exploratory and speculative.

We found that 25% of the articles were difficult to read due to an unclear definition of the primary response or outcome variable. Although it is valuable for medical studies to evaluate several aspects of patients' responses, it is important to identify a small set of primary outcome or response variables in advance [16]. It is also important that the results for primary responses (including any non-significant findings) are fully reported [9]. Focusing on clearly stated primary response measure(s) helps both the investigators to write an understandable and compact report and the readers to evaluate the findings. Again, though, our results indicate that having an unclear primary response or outcome variable does not lower the citation count and so does not appear to restrain other researchers from using the paper.

Articles with clearly documented research methods did receive more citations. This association was more marked in papers published in AJP and BJP. In our sample, documentation of statistical methods used was generally sufficient in AGP (92.2%), consistent with the editorial policy of the journal which requires an extended methods section in submitted manuscripts.

We included in our review four general psychiatric journals with different prestige and visibility. By involving several journals we were able to control for the effect of journal visibility on the number of citations received and compare the prestige of a journal with the quality of statistical presentation. The reporting of statistical information was more detailed, comprehensive and useful for the reader in the two leading journals (AGP and AJP). Again, this is consistent with their detailed guidelines for presenting statistical results, and also a more rigorous review process, including extensive statistical reviewing [17]. In low-impact journals the peer review is undoubtedly less thorough [6,18]. Thus our results provide an important confirmation, for editors, authors and consumers of research, on the value of guidelines and rigorous statistical reviewing.

Several findings have demonstrated that a non-negligible percentage of articles – even those published in 'high -prestige' journals –, are not statistically faultless [6,8,19,20]. Our findings are in line with these studies, and also demonstrate inadequate reporting of research methods and hypotheses. However, most of the statistical problems in medical papers are probably relatively unimportant or more a matter of judgment. As there is also no general agreement on what constitutes a statistical error, the comparison of different statistical reviews is difficult [8,21]. There may be several valid ways of analyzing a data set.

It has been claimed that researchers prefer to cite large studies rather than small studies [22]. Our data does not support this hypothesis: sample size was not associated with the frequency of citations. Callaham et al [15] came to the same conclusion when they analyzed a set of emergency medicine articles. Textbooks of medical statistics require that the sample size should be large enough (or as large as possible) and that some justification for the size chosen should be given [23]. Unfortunately, our results suggest the concept of sample size calculations seems to be almost unknown in psychiatric research outside the field of clinical trials; less than 4 % of the evaluated articles included sample size calculations, power analysis or any other justification for the sample size.

## Conclusion

In this cohort of published research, measures of reporting quality and appropriate statistical analysis were not associated with the number of citations. The journal in which a study is published appears to be as important as the statistical reporting quality in ensuring dissemination of published medical science [2,24]. A highly visible publication may therefore attract more attention, even if the results are poorly and obscurely reported. Thus, the quality of statistical reporting is often not important in the subsequent update of an article. Rather, if a study is highly cited it reflects a strong active interest in the question addressed in the scientific community [25].

Most of the errors and shortcomings in the application and reporting of statistical information in the journal articles reviewed here are related to topics included in most introductory medical statistics books. Some of these errors are serious enough to call the author's conclusions into question. It seems strange that a problem seemingly so important, so wide spread and so long-standing should continue [6,9]. Possible explanations are that (1) much research is done without the benefit of anyone with adequate training in quantitative research methods [26], (2) copying of inappropriate methods is usual [8] or (3) the statistical component of the peer review process is not common or sufficiently valued by editors [17]. Our study suggests another possible contributory factor. Editors and authors are often partially motivated by the desire to publish papers that will be highly cited and, while the methodological quality of published original research articles does not appear to relate to their uptake in the literature, poor reporting and errors in the analysis are likely to continue.

## Competing interests

The author(s) declare that they have no competing interests.

## Authors' contributions

PN and MS had the idea for the article. PN collected data, did the statistical analysis and wrote the paper. GR contributed to the data collection, the statistical analysis and writing of the paper. JC contributed to the statistical analysis and writing of the paper. MS initiated the study project, coordinated the collection of material and contributed to the writing of the manuscript. All authors read and approved the final manuscript.

## Pre-publication history

The pre-publication history for this paper can be accessed here:


